# 2169. A 4-Year Analysis of Antimicrobial Susceptibility to Ceftolozane/Tazobactam in *Pseudomonas aeruginosa*: How Drug Availability Affects Resistance Rate

**DOI:** 10.1093/ofid/ofad500.1791

**Published:** 2023-11-27

**Authors:** Alessandra Imeneo, Laura Campogiani, Andrea Di Lorenzo, Cartesio D’Agostini, Anna Altieri, Vincenzo Malagnino, Massimo Andreoni, Marco Iannetta, Loredana Sarmati

**Affiliations:** Tor Vergata University of Rome, Rome, Lazio, Italy; Tor Vergata University of Rome, Rome, Lazio, Italy; Tor Vergata University of Rome, Rome, Lazio, Italy; Tor Vergata Hospital, Rome, Lazio, Italy; Tor Vergata Hospital, Rome, Lazio, Italy; Tor Vergata University of Rome, Rome, Lazio, Italy; Tor Vergata University of Rome, Rome, Lazio, Italy; Infectious Diseases Unit, Tor Vergata University of Rome, Rome, Rome, Lazio, Italy; Tor Vergata University of Rome, Rome, Lazio, Italy

## Abstract

**Background:**

Antibiotic resistance is associated with a fitness cost and reduced bacterial growth that allows susceptible bacteria to outcompete resistant bacteria, if selective pressure from antibiotics is reduced. Ceftolozane/tazobactam (C/T) approved in 2014, was temporarily withdrawn from December 2020 to February 2022: this forced unavailability created the conditions to study how drug discontinuation might influence resistance reversibility.

**Methods:**

This is a retrospective observational study, including isolates of *P. aeruginosa* collected between March 1^st^ 2019 and February 22^nd^ 2023. Isolates collected from swabs (e.g. rectal, skin) or with no C/T susceptibility test were excluded. Antibiotic susceptibility tests were performed with the Vitek-2 assay. Only the first isolate for each patient was included; multiple isolates from the same patient were included if collected at least 5 months apart. Four collection periods were defined (Fig 1). All data were analyzed using JASP.

**Figure:**
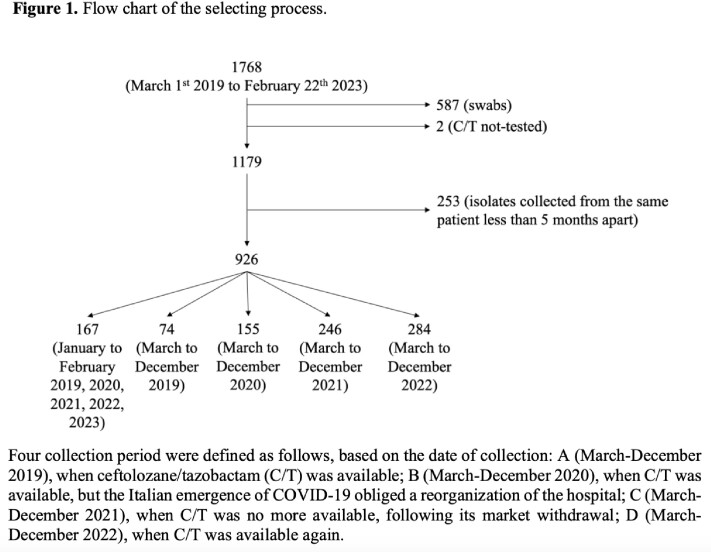

**Results:**

From the Hospital microbiology laboratory list, 1768 *P. aeruginosa* isolates were derived, of which 926 isolates fulfilled the inclusion criteria. A significant (p-value < 0.001) reduction of C/T resistance rate was observed when the antibiotic was unavailable, followed by a subsequent increase with its reintroduction (Tab.1). Evaluating phenotypic susceptibility to other antibiotics, production of GES-variants, suggested by resistance to carbapenem and C/T but retained susceptibility to ceftazidime/avibactam, was most likely the predominant resistance mechanism (Tab. 2); a reduction in GES-like isolates, concurrent with decreasing C/T resistance was observed. 19 patients presented a C/T-resistant isolate after a previous susceptible one, but only 4 patients had received a prior C/T treatment (Tab 3).

**Figure:**
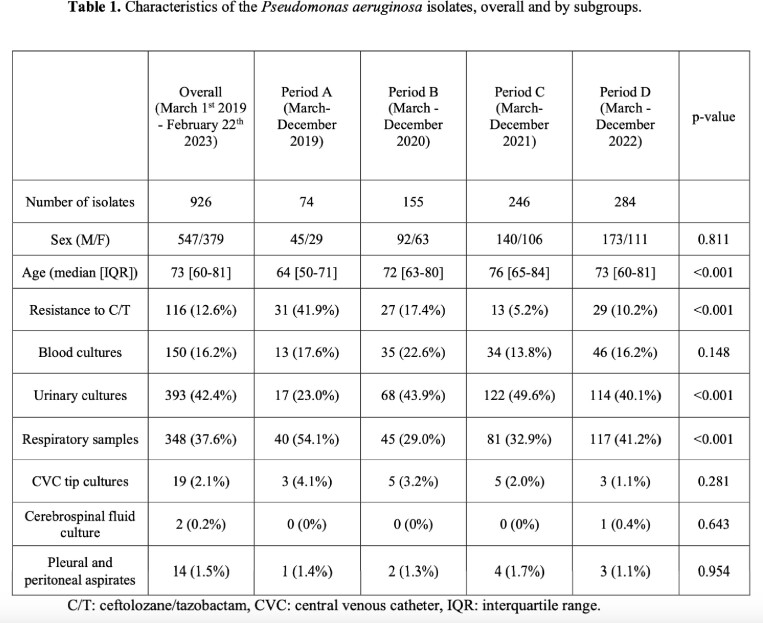

**Figure:**
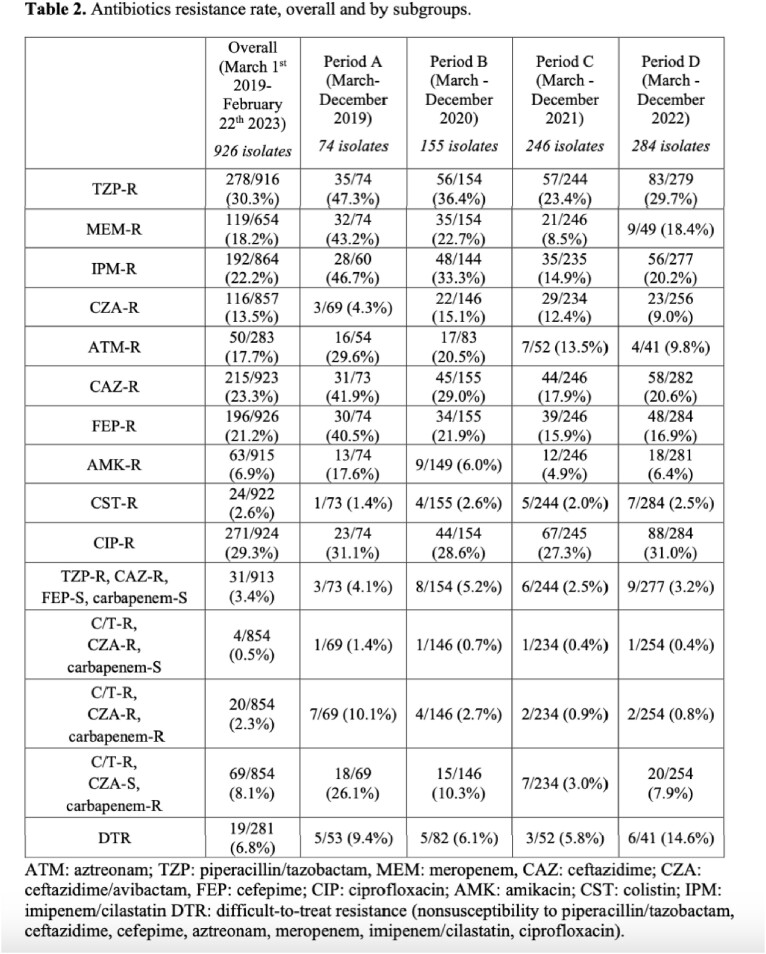

**Figure:**
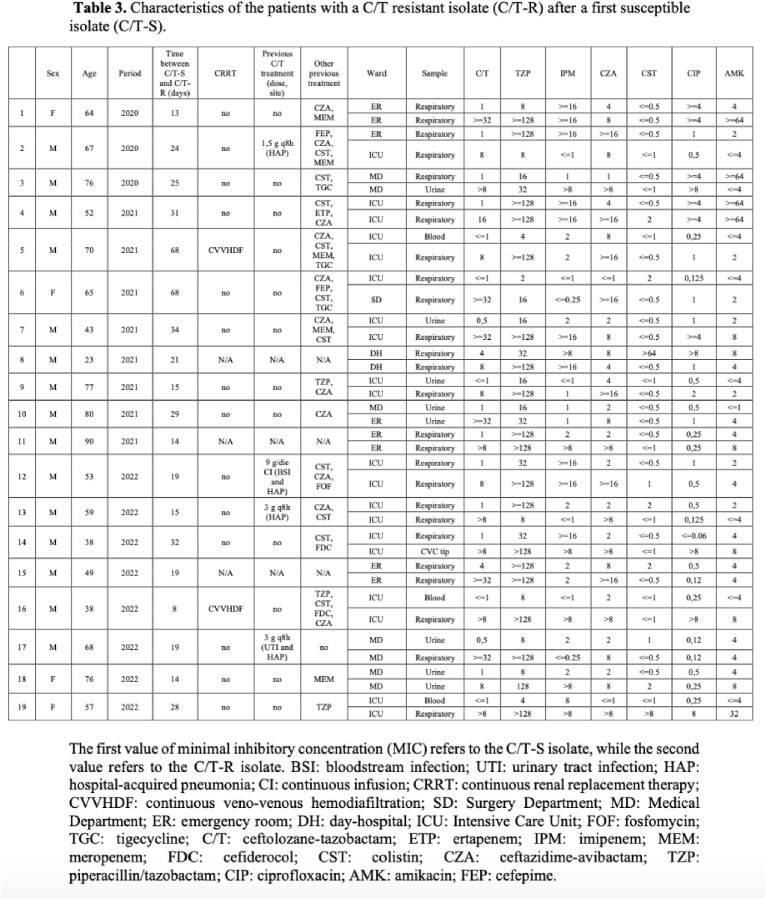

**Conclusion:**

The temporary unavailability of C/T created the condition to analyze the practical application of the theory of fitness cost to maintain resistance. Our data show a significant reduction of resistance in the absence of the antibiotic, but a subsequent increase with its reintroduction, due to the persistence of resistant isolates. Therefore, continuous monitoring of antibiotic use and evolving resistance is essential.

**Disclosures:**

**All Authors**: No reported disclosures

